# Prevalence and predictors of overweight and obesity among school-aged children in urban Ghana

**DOI:** 10.1186/s40608-017-0174-0

**Published:** 2017-12-04

**Authors:** Richmond Aryeetey, Anna Lartey, Grace S. Marquis, Helena Nti, Esi Colecraft, Patricia Brown

**Affiliations:** 1 School of Public Health, University of Ghana, Box LG 13 Legon, Accra, Ghana; 2 Department of Nutrition and Food Science, University of Ghana, Box LG 134 Legon, Accra, Ghana; 3 School of Dietetics and Human Nutrition, McGill University, 21,111 Lakeshore Road, Ste-Anne-de-Bellevue, Montreal, QC H9X 3V9 Canada; 4 Department of Biochemistry and Biotechnology, Kwame Nkrumah University of Science and Technology, Kumasi, Ghana

**Keywords:** School-age children, Overweight, Obesity, Physical activity, Urban, Ghana

## Abstract

**Background:**

Childhood overnutrition is a serious public health problem, with consequences that extend into adulthood. The aim of this study was to determine the prevalence and determinants of overweight and obesity among school-age children in two urban settings in Ghana.

**Methods:**

This cross-sectional study involved 3089 children (9–15 years) recruited between December 2009 and February 2012 in Accra and Kumasi, Ghana. Socio-demographic, dietary, and physical activity data were collected using pretested questionnaires. BMI-for-age z-scores were used to categorize anthropometric data of the children as thin, normal, or overweight/obese. Determinants of overweight were examined using multiple logistic regressions.

**Results:**

Seventeen percent of children were overweight or obese. Children who reported lower participation (< 3 times/week) in sports activity were 44% more likely to be overweight or obese (AOR = 1.44; 95% CI: 1.07, 1.94). Maternal tertiary education (AOR = 1.91, 95% CI: 1.07, 3.42), higher household socioeconomic status (AOR = 1.56, 95% CI: 1.18, 2.06), and attending private school (AOR = 1.74, 95% CI: 1.31, 2.32) were also associated with elevated risk of overweight and obesity.

**Conclusions:**

Physical inactivity is a modifiable independent determinant of overweight or obesity among Ghanaian school-aged children. Promoting and supporting a physically active lifestyle in this population is likely to reduce risk of childhood overnutrition.

## Background

Childhood overweight and obesity is a serious public health challenge affecting both developed and developing countries [1]. The prevalence of overweight and obesity is increasing rapidly in developing countries; in some countries, high rates of childhood overweight (> 15%) have been reported [2]. The current increasing prevalence of overweight has been partly attributed to the nutrition transition which is characterised by systemic societal changes such as increased urbanization, industrialization, trade liberalization, and economic growth. All these changes influence the food system in ways that then fuel behavior changes linked with increased energy-dense food consumption and reduced physical activity [3, 4]. In particular, living in an urban setting has been linked with increased risk of childhood obesity in developing countries [2, 5].

One suggested pathway through which urbanization influences overnutrition is by reducing opportunities for physical activity [6]. The reported mechanisms of this relationship include increased access to and use of motorized transport [7, 8] as well as computerized devices which displace time which would otherwise be used for activity [9, 10]. Simultaneously, urban-dwelling children do not consume adequate amounts of fruits and vegetables, and also have more access to energy-dense foods high in fat, sugar and salt, including out-of-home, ready-to-eat meals and snacks [11]. In urban Benin, out-of-home prepared foods contributed more than 40% of the daily energy intake of school-going adolescents; those who consumed more than 55% of energy out of home ate more sweetened energy-dense foods, and less fruits and vegetables compared to those who consumed less out of home (< 34% of energy) [12].

Childhood and adolescent overweight and obesity are associated with both short- and long-term adverse effects related to health and development. In the short term, obese young adolescents have an elevated risk of low self-esteem, negative self-image, hyperlipidemia, elevated blood pressure, and hyperinsulinemia compared to non-obese children [13, 14]. In addition, overweight in early childhood is likely to persist into adulthood, and thereby further increase risk of overweight-related chronic disease sequelae in adulthood [15]; this relationship is particularly stronger among older children (>10 years) [16, 17]. Thus, childhood overweight is associated with adverse effects on adult outcomes resulting in an unhealthy workforce, increased cost of health care, and limiting total population productivity. It is therefore important that strategies to address overweight and obesity start among children and adolescents. A critical step towards addressing overweight is a better understanding of the scope of the problem, as well as associated factors.

In Ghana, information on childhood obesity is scarce, particularly for children of school age. The 2007 Global School-based Student Health Survey reported overweight and obesity prevalence of 7% among Ghanaian children 13–15 years [18]. This survey is, however, limited by its use of self-reported anthropometric data among both rural and urban school-going children. There is thus a gap in knowledge on the magnitude and determinants of overweight and obesity among school-going children that is based on a representative sample of the urban population. Identifying risk factors of overnutrition among children and adolescents will provide the basis for comprehensive interventions to address obesity. Therefore, the main objective of this study was to determine the prevalence and risk factors of overweight and obesity among 9–15 year old school-age children in two urban settings of Accra and Kumasi in Ghana. The study will also explore the key risk factors of overweight and obesity among school-age children in Ghana.

## Methods

### Study population

**Fig. 1:**
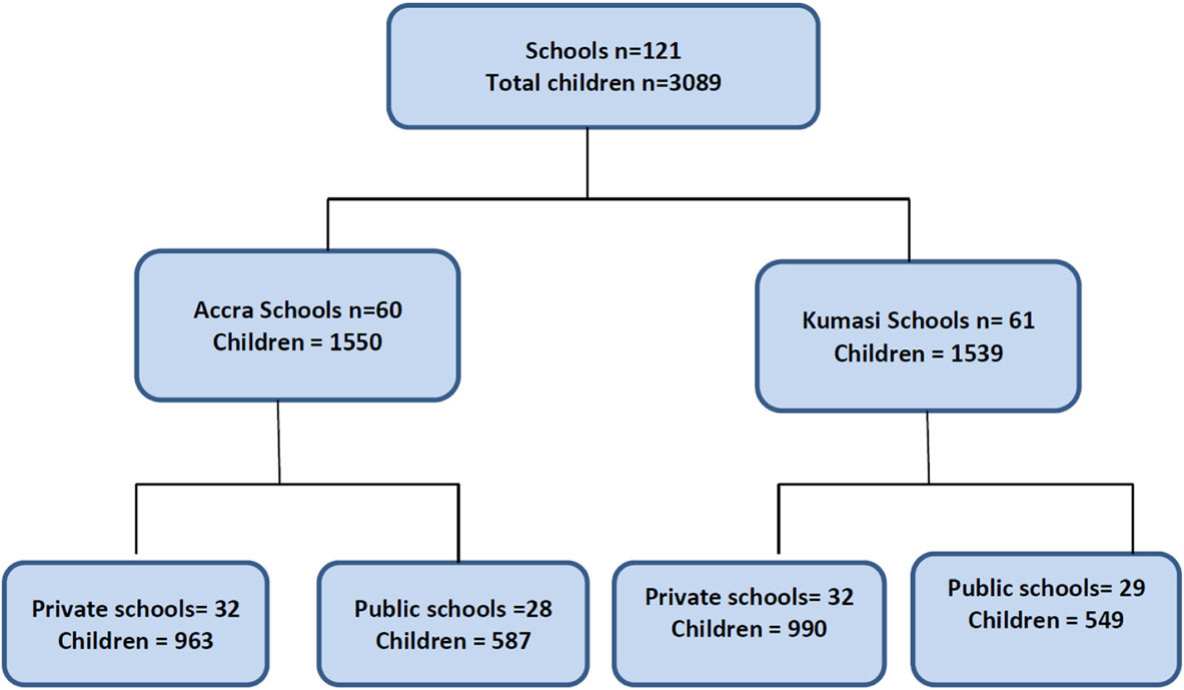
Diagram showing flow of school children in the study

This was a cross-sectional survey involving 3089 school-age children between the ages of 9 and 15 years who were recruited from 121 schools located in the two largest urban centres of Ghana: Accra (the capital city of Ghana) and Kumasi (Fig. 1). Children in the 9–15 years age group were recruited from either upper primary level or junior high school level. This age group was selected for two main reasons: 1) under-representation in nation-wide surveys, and 2) it is a target of on-going school nutrition interventions in the Ghanaian context. The schools included in the study were either exclusively primary, exclusively junior high, or having both primary and junior high level children together in a single school. The study was implemented between December 2009 and February 2012.

The study was approved by the Ethical Review Boards of McGill University (A09-B21-09A), Canada and the Noguchi Memorial Institute for Medical Research (004/09–10), University of Ghana, Legon. Prior to data collection, administrative permissions were also obtained from the national office of the Ghana Education Service as well as from head teachers of all participating schools. Written informed consent was obtained from all parents whose children participated in the study. In addition, each participating child provided signed assent before the questionnaire was administered.

### Sampling

Due to expected higher prevalence of overweight and obesity among children attending private schools in Ghana, sample sizes were estimated separately for public and private schools. In public schools, the estimated sample was 954; in private schools, the estimate was 1808. These estimates were based on an overweight prevalence of 10% in private schools and 5% in public schools, a margin of error of 1.5%, and a 95% confidence interval, and allowing for 15% loss due to incomplete data. Using a cluster sampling plan, 57 public schools that had both primary and junior high school (JHS) departments were randomly selected. Assuming 20% parental refusal, a total of 20 pupils (10 males and 10 females) were randomly selected and contacted in each school. Using a similar cluster sampling for the private schools, 64 private primary and junior secondary schools were randomly selected. In each school, 36 pupils (18 females and 18 males) were randomly selected and contacted.

### Data collection

Questionnaires were administered to the school children, individually. Data collected with the questionnaire included socio-demographic characteristics, dietary habits, physical activity, and television viewing. Each child and parent had the weight and height measurements taken and recorded by a trained research assistant. The measurements were taken at the school premises.

### Socio-demographic data

A structured pre-tested questionnaire was used to collect information on household demographic and socio-economic characteristics including educational, home living arrangements, and occupation of parents. In addition, ownership of household assets, including refrigerator and television, video player, and automobile were documented. This information was obtained from the children with the assistance of parents (where a parent was available).

### Dietary and physical activity assessment

Dietary intakes of the school children were assessed using a food frequency questionnaire that had a reference period of one week prior to the survey. The questionnaire consisted of 60 food items and focused on describing patterns of consumption of high fat foods, high sugar foods, sweetened drinks, fruits, and vegetables. The frequencies of intake of the listed foods over time (daily and weekly) by the school children were then determined. The food frequency questionnaire was designed for this study by identifying commonly consumed foods in Ghana under each of 11 food groups. The food groups were sugar-sweetened beverages, milk and dairy products, cereal products (including breads and biscuits), fried foods, animal-source foods, spreads and toppings, fruits, vegetables, soups, sweets and high calorie foods, and other staple foods. An initial list of foods was pre-tested among mothers in Accra, following which additional foods were added. During the survey, opportunity was provided for including additional foods that were reportedly consumed. A pre-tested questionnaire was used to collect information on the level of physical activity and sports participation of study children. The specific questions included the frequency and duration of television viewing, number of days per week child walked to school, and frequency of performing house chores and participation in sporting and other physical activities including football, *ampe* (indigenous Ghanaian jumping game), hockey, table tennis, lawn tennis, rope skipping, volleyball, basketball, swimming and gardening.

### Anthropometry

All anthropometric measurements were carried out at the school premises. Participants removed all heavy clothing and accessories (such as shoes or sandals, belts, watches, and sweaters) and emptied their pockets (where necessary), prior to the measurement. Body weight was measured to the nearest 0.1 kg using the Tanita Digital Scale (model BWB-800, Tanita Corporation, USA). Height measurements were taken to the nearest 0.1 cm using the Shorr Board (Shorr Productions, Olney, MD). Parents were invited to the school for weight and height to be taken. All measurements were done and recorded in duplicate. Weight and height measurements were converted to body mass index for age z-scores (BMIZ) based on the WHO Child Growth Standards [19]. Overweight was defined as BMIZ greater than one standard deviation from the median; obesity was determined as BMIZ greater than two standard deviations [20].

### Statistical analyses

Two factors were created from a set of seven socio-economic status (SES) variables using factor analysis with varimax rotation as proxy indicators for household socio-economic status. The first factor reflected household items such as television and refrigerator and the second reflected occupation and ownership of items such as home, air conditioner, and vehicle. Tertiles of the factors are reported. Regarding dietary data, proportions were reported for how frequently dietary behaviors and foods with established links to obesity were reported by respondents. Analyses were carried using cases with complete data. The proportion of children who were overweight or obese (BMIZ >1 SD) was computed. Multiple logistic regression procedure was used to examine characteristics that were statistically and independently associated with overweight or obese status. The factors considered were those that were shown to be either significantly correlated (*p* < 0.05) or tended to be correlated (*p* < 0.10) with overweight and obesity, and included child characteristics (age, sex, dietary habits, physical activity, type of school), maternal characteristics (education, occupation), and household characteristics (household wealth status). The region of residence and correlation within clusters (school) were controlled for in the model. The final model included only factors that were associated with overweight or obesity at *p* < 0.05. We used weights in the analysis to restore the representativeness of the sample. All statistical analyses were conducted using SAS (version 9.2, Cary, NC, USA) and statistical significance in the final model was determined at *p* < 0.05.

## Results

The current analysis included 3089 out of the 3444 school children who were sampled (Fig. 1). The majority (90%) of children who were sampled but not included did not show up on the day of data collection; the remainder either refused participation (9%) or were ineligible because of their age (1%). The mean age of children who participated in the study was 12.2 ± 1.7 years and more than half of them were female (Table 1). Most of the children ate breakfast during the school week, with 85% having breakfast more than three days per week (Table 2). Consumption of fruits and vegetables was low. Only 20% and 38% had consumed fruits and vegetables >5 times, respectively, the previous week. About three-quarters of the children (76%) walked to school at least four out of the five school days in a week and more than half (58%) did household chores during the week. However, involvement in sporting activities was low, with less than one-third of the children engaging in a sport at least three times in a week. Television watching was also low among the study sample. Less than 15% watched television at least five times during the week prior to the survey. The overall prevalence of overweight and obesity was 14.7% among the children, with 4.4% being obese (Table 3). A higher proportion of children were overweight (including obese) in the private compared to the public schools (21.4% vs 11.2%, *p* < 0.001).

**Table 1:** Background characteristics of Ghanaian children 9–15 years

	Total		Private School		Public School	
	n	%	n	%	n	%
*Child’s sex*						
Male	1413	46.6	925	51.1	488	46.7
Female	1617	53.4	1028	48.9	648	53.3
*Maternal education*						
None	174	56	74	3.8	100	8.8
Primary	1055	34.2	558	28.0	497	43.5
Secondary (JHS/SHS)	637	20.7	458	23.3	179	15.7
Tertiary	357	11.6	287	14.8	70	6.1
Do not know	860	27.9	572	30.1	288	25.9
*Maternal occupation*						
Artisan	515	16.7	302	15.5	213	18.8
Professional^a^	380	12.3	302	15.5	78	6.7
Office worker^b^	71	2.3	52	2.6	19	1.7
Trading	1884	61.0	1140	58.4	744	65.5
Not employed	195	6.3	126	6.4	69	6.2
Do not know	44	1.4	31	1.6	13	1.1
*Household size*						
≤ 3	309	10.0	200	10.2	109	9.5
4–6	1785	57.8	1121	57.7	664	58.8
7–9	810	26.2	505	25.7	305	26.6
≥10	185	6.0	127	6.4	58	5.1
*Household socioeconomic status factor 1* ^c^						
Low	1027	33.7	575	30.0	451	40.3
Medium	981	32.2	725	37.7	256	22.6
High	1037	34.1	621	32.3	416	37.1
*Household socioeconomic status factor 2* ^d^						
Low	1199	39.4	603	31.3	596	53.3
Medium	825	27.1	501	26.1	324	28.9
High	1021	33.5	818	42.6	203	17.7

Values presented as number (percentage of private or public)

^a^Includes teachers, lawyers, doctors, and accountants

^b^Includes secretaries and office clerks

^c^Reflects possession of household items such as television, video player, and refrigerator

^d^Reflects occupation and ownership of assets such as home, air conditioner, and vehicle

**Table 2:** Dietary and physical activity habits of Ghanaian children 9–15 years

	Total		Private		Public	
	n	%	n	%	n	%
*Dietary habits*						
Access to soft drinks at home	860	27.8	626	32.1	234	20.3
Breakfast ≤3 days/week	427	13.8	289	14.9	138	12.1
Fried foods ≥5 times/week	1388	44.9	728	37.3	460	40.5
Soft drinks ≥2 bottles previous day	33	1.1	24	1.3	9	0.8
Sweetened drink ≥5 times/week	465	15.1	331	16.9	134	11.8
Cakes, pies, doughnuts ≥3 days/week	1742	56.4	1071	54.5	671	58.6
Fruit consumption (times/week)						
0–5	2464	79.8	1563	80.0	901	79.7
6–10	452	11.7	282	14.4	170	14.6
11–15	138	3.6	89	4.6	49	4.3
> 15	35	0.9	19	1.0	16	1.4
Vegetable consumption (times/week)						
0–5	1899	61.5	1210	62.6	689	60.7
6–10	864	30.0	550	27.7	314	27.6
11–15	236	7.6	138	6.9	98	8.6
> 15	90	2.9	55	2.8	35	3.1
*Physical activity*						
Transport to school ≥3 days/week	1347	43.6	1034	52.9	313	27.2
Household chores >5 times/week	1795	58.1	1041	53.3	754	65.7
Any sporting activity ≥3 times/week	852	27.6	498	26.2	354	32.0
Playing football/ampe^a^ ≥ 3 times/week						
Males	578	18.7	349	9.2	229	21.9
Females	275	8.9	149	7.0	125	10.1
*Sedentary behavior*						
Watching Television ≥5 times/week	166	5.4	105	13.9	61	13.7
Duration watching TV (hours/week)						
<2	1356	45.1	854	45.1	502	45.2
2–3	1356	45.1	856	45.2	500	45.1
≥4	291	9.8	183	9.7	108	9.7

Values presented as number (percentage of private or public)

^a^A local game involving clapping and jumping

**Table 3:** Nutritional status of Ghanaian School children ages 9–15 years

Growth status	Total		Private		Public	
	n	%	n	%	n	%
Thin	102	3.3	55	2.9	47	4.4
Normal	2429	78.6	1475	75.7	954	84.3
Overweight	382	12.4	282	14.2	100	8.3
Obese	143	4.6	113	5.8	30	2.5
Severely obese	33	1.1	28	1.4	5	0.4
Stunting	99	3.2	50	2.6	49	4.4

Thin: BAZ < −2SD; Overweight: +1SD < BAZ ≤ +2SD; Obese: +2SD ≤ BAZ ≤ +3SD; Severely Obese: BAZ > +3; Stunting: HAZ < −2SD; (WHO, 2007)

### Risk factors of overweight and obesity

Table 4 shows the factors that were significantly associated with being overweight or obese in the study sample, based on multiple logistic regression. Female children were twice as likely to be overweight or obese compared to male children (AOR = 2.38, 95% CI: 1.79, 3.18). None of the dietary habits that were assessed was significantly associated the risk of overweight or obesity. Physical activity was a determinant of overnutrition among the children. Children who engaged in sports for less than three times a week were at a 44% higher odds of being overweight or obese when compared to those who were involved in sporting activities at least three times a week. High maternal education and household SES were risk factors for overweight and obesity. Children of mothers who received formal education beyond the secondary level were more likely to be overweight or obese compared to those whose mothers had no education (AOR = 1.91, 95% CI: 1.07, 3.42). However, being educated up to the secondary level was not linked with overweight. Children living in households in the third SES tertile had 56% higher odds of being overweight or obese when compared to those from households in the first tertile (lowest SES). After adjusting for biologic factors, dietary and physical activity habits, and SES, those attending private schools were more likely to be overweight or obese compared those who attended public schools (AOR = 1.74, 95% CI: 1.31, 2.32).

**Table 4:** Factors associated with overweight and obesity (BMIZ >1 SD) among Ghanaian children 9–15 years

	Adjusted Odds Ratio^b^	95% Confidence Interval	*p*-value
*Child’s sex*			
Female	2.38	1.79, 3.18	<0.01
Male	1		
*Breakfast ≥ 3 days/week*			
No	0.76	0.58, 1.00	0.05
Yes	1		
Eats Cakes, pies, doughnuts ≥3 days/week			
Yes	0.83	0.66, 1.04	0.10
No	1		
*Fruit consumption (frequency/week)*			
> 15	0.41	0.14, 1.17	0.09
11–15	1.13^a^	0.65, 1.93	0.67
6–10	1.07^a^	0.78, 1.46	0.69
0–5	1		
*Vegetable consumption (frequency/week)*			
> 15	1.27	0.69, 2.32	0.44
11–15	1.48	0.99, 2.23	0.06
6–10	1.16	0.92, 1.46	0.20
0–5	1		
*Transported to school (days/week)*			
4–5	1.39	1.06, 1.82	0.02
1–3	1.11	0.52, 2.37	0.79
Never	1		
*Engaged in any sporting activity ≥ 3 times/week*			
No	1.44	1.07, 1.94	0.02
Yes	1		
*School type*			
Private	1.74	1.31, 2.32	<0.01
Public	1		
*Maternal education*			
Tertiary	1.91	1.07, 3.42	0.03
Secondary	1.00	0.57, 1.75	0.99
Primary	1.12	0.68, 1.84	0.65
Don’t know	1.14	0.69, 1.89	0.61
None	1		
*Household socioeconomic status*			
High	1.56	1.18, 2.06	<0.01
Medium	1.10	0.81, 1.49	0.54
Low	1		

^a^Borderline significant values (0.05 ≤ *P* < 0.08)

^b^Other variables controlled in the analysis: age, child engaged in household chores, and frequency of sweetened beverage consumption

## Discussion

Among Ghanaian school children 9–15 years living in Accra and Kumasi, the prevalence of overweight (including obesity) was 15%. Fundamentally, overweight and obesity reflects positive energy balance; physical inactivity and poor dietary habits are two key modifiable factors that can influence this balance in a population. In the current study, low physical activity participation was associated with overweight and obesity among school-going children in urban Ghana, similar to earlier studies in other developing countries [2, 21, 22]. Both low participation in structured (sports) and unstructured forms of physical activity (e.g., walking to school) were related to overweight and obesity, indicating the need to encourage varied opportunities for physical activity among school-aged children.

In line with the WHO global strategy on diet, physical activity, and health, the Ministry of Health in Ghana recommends that children and adolescents have at least one hour of moderate to vigorous physical activity daily, and that physical education (PE) of not less than 2 h per week should be included in the school curriculum [23, 24]. Although PE is part of the basic education curriculum, studies show that the main focus has been on competitive sports [25]. While PE aims to encourage majority of students to participate regularly in physical activities, competitive sports is described as a value-added experience for few students who show the potential for elite performance in specific structured activities. Thus, focusing mainly on competitive sports in school limits opportunity for the majority of children to engage in school-based physical activity.

Parental work habits is known to influence the level of physical activity among children [26]. When parents work mostly away from home, as is commonly observed in urban settings, there is limited time to supervise or engage in recreational activities with their children. Children are thus left on their own to decide the use of the period after school. With the upsurge of video and computer games and television stations, children are likely to engage in sedentary activities that involve spending time in front of a screen, thereby limiting their opportunities for engaging in moderate and vigorous physical activities. An earlier study in the Ga-East district of Ghana reported that obese and overweight school children (8–18 years) spent more time watching television and playing video games (90 min/day) than engaging in physical activities (50 min/day) [27]. In the current study, more than half of children spent at least two hours/day watching television in the week prior to the survey.

The positive association between SES and overweight (including obesity) observed in this study is similar to studies in other developing countries [2, 21, 28, 29]. The direction of the association between obesity and SES varies from positive in poorer countries to negative among better-off societies [30]. Households with high SES may have more access to and be able to afford processed, fatty, and/or sugary foods and beverages compared to poorer households. Socio-Economic Status may also predict access to technology (e.g., television, cars, computers, and video games). These technology devices are likely to contribute to a more sedentary lifestyle. In our study, however, SES was associated with overweight and obesity independent of physical activity. Thus, there may be other factors that mediate the observed relationship.

Available studies on the relationship between maternal education and overweight/obesity in children and adolescents are mixed. While some studies found a positive association [28, 31], others have reported a negative [32, 33] or no [21] association. In the present study, children whose mothers had received post-secondary education were more likely to be overweight or obese compared to those who had no formal education. High level of maternal education may lead to improved acquisition and use of nutrition knowledge which can translate into good dietary practices [34]. On the other hand, mothers with higher levels of education are likely to earn higher income. The latter has been linked with adverse affects on dietary and/or physical activity habits through the easier accessibility of energy-dense foods and electronic devices that promote sedentary lifestyles. Thus, the relationship between maternal education and overnutrition among children may be modified by other factors and therefore needs further investigations.

Previous studies have established a strong association between diet and risk of overweight in both developed and developing country settings [35–37]. In the current analysis, however, there was no statistically significant association observed between overweight and any of the indicators of dietary behavior. This can be explained by both the inherent imprecision of measuring diet by recall, as well as the detail of dietary analysis reported in the current analysis. It is important, however, that this finding is neither misunderstood nor misrepresented as evidence of the association between overweight and diet. Subsequent analysis of the dietary data will enable better understanding of the links between diet and overweight as well as other biological outcomes examined (including lipid profile).

One of the strengths of this study was the use of the WHO growth reference for school-aged children and adolescents in the determination of overweight and obesity. Compared to the previous NCHS growth curves [38], the WHO reference curves are closely aligned with Child Growth Standards at five years of age as well as the recommended adult cut-offs for overweight and obesity at 19 years, which is the WHO upper age limit for adolescence [20]. It provides, therefore, an appropriate reference for the age group that participated in the current study. Further, the study included more than one hundred schools that were located throughout the two largest cities in Ghana. The use of random sampling to select the children enhanced our sample as representative of the school-going children in urban settings in Ghana.

The findings of the current study should be interpreted bearing in mind its inherent limitations. First, we recognise the inability of this study to establish causality due to its cross-sectional design. Additionally, although parental BMI has been shown to be a risk factor for overweight and obesity among children and adolescents [5, 32, 33, 39], this could not be controlled for in the regression analyses. This was due to the lack of anthropometric data for more than 20% of parents who were interviewed by phone. Finally, and importantly, the dietary information was collected using food frequency questionnaire and thus any interpretations from the diet-related analyses should be viewed with this inherent limitation in mind.

## Conclusions

In conclusion, the prevalence of overweight and obesity among school-going children living in urban areas in Ghana was high. This study identified physical activity status, sex of child, maternal education, household SES, and type of school as significant determinants of overweight and obesity in these children. Of these factors, physical activity is the one that can be modified among school-going children. The current physical education curriculum for basic schools in Ghana needs to be re-assessed and updated to encourage more children and adolescents to participate regularly in physical activities. Further, there is need to champion the utilization of the PE time for physical activity in schools. This may require placing more emphasis on non-competitive activities. In addition, school children should be encouraged to walk to school as much as possible and this recommendation should be supported by policies that ensure safety on walk routes.

## Abbreviations

AOR: Adjusted Odds Ratio
BMI: Body Mass Index
BMIZ: Body Mass Index Z-Score
CI: Confidence Interval
GSHS: School-based Student Health Survey
JHS: Junior High School
PE: Physical education
SD: Standard Deviation
SES: Socio-Economic Status
WHO: World Health Organization
